# An Efficient Cross-Modal Interaction and Dynamic Fusion Network for Multimodal Breast Ultrasound Diagnosis

**DOI:** 10.3390/tomography12070093

**Published:** 2026-06-25

**Authors:** Xiangqiong Wu, Yin Lan, Lina Han, Peng Wang

**Affiliations:** 1 School of Computer Science, Hunan First Normal University, Changsha 410205, China; wuxiangqiong@hnu.edu.cn; 2 School of Electronic Information, Hunan First Normal University, Changsha 410205, China; 23414030308@hnfnu.edu.cn (Y.L.); 23414030310@hnfnu.edu.cn (L.H.)

**Keywords:** breast ultrasound, cross-modal attention, feature enhancement, dynamic fusion

## Abstract

**Background:** Multimodal breast ultrasound, including B-mode imaging, color Doppler flow imaging, and elastography, provides complementary information for lesion characterization. However, effectively integrating heterogeneous modalities remains challenging due to inconsistent feature distributions, limited cross-modal interaction, computational cost in existing methods, and sensitivity to noise and missing data. **Methods:** We presented an efficient Cross-Modal Interaction and Dynamic Fusion Network (CIDFNet) for multimodal breast ultrasound analysis. The framework integrates a multi-scale feature enhancement module to improve modality-specific representations, a cross-modal interaction module to enable early-stage feature exchange across modalities, and a dynamic fusion strategy to adaptively combine modality information based on feature reliability estimation. In addition, an invertible neural network is incorporated to reconstruct missing modality features during training. **Results:** Experiments on an internal dataset of 248 patients with 1532 images show that CIDFNet obtains an AUC of 85.69%, accuracy of 75.51%, recall of 50.00%, F1-score of 62.50%, and precision of 83.33%, while requiring 49.51 M parameters and 79.79 G FLOPs, respectively. Under a simplified Gaussian noise perturbation setting, performance degradation is observed. **Conclusions:** CIDFNet presents a framework for multimodal breast ultrasound analysis that reflects a trade-off between performance and computational efficiency.

## Simple Summary

Multimodal breast ultrasound provides complementary information for lesion characterization, while effective integration remains challenging due to heterogeneous feature distributions, limited cross-modal interaction, and sensitivity to noise and missing data. This study presents an efficient framework that enhances modality-specific features, enables early-stage cross-modal interaction, and adaptively fuses multimodal information. The proposed method reduces computational cost while achieving high area under the curve, and shows reduced performance variation under noisy test conditions. These results provide preliminary insights that may support further research in multimodal medical imaging.

## 1. Introduction

**Figure 1:**
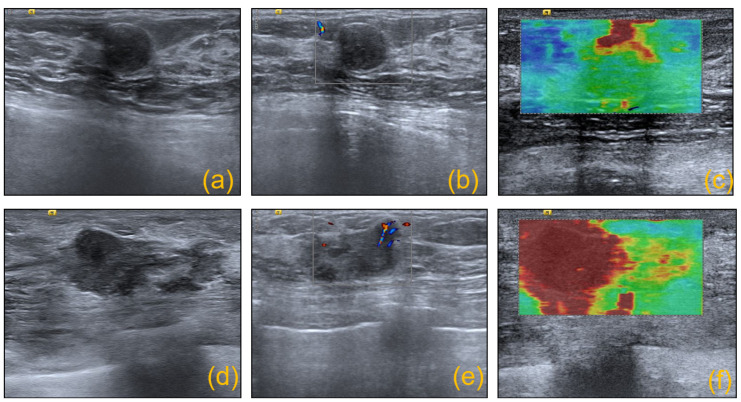
Multimodal ultrasound examples of benign (**top row**) and malignant (**bottom row**) breast lesions. (**a**,**d**) BUS; (**b**,**e**) CDFI; (**c**,**f**) UE.

Breast cancer remains one of the most common malignancies among women globally. According to 2022 global cancer statistics [1], the disease accounted for approximately 2.3 million new cases and 680,000 deaths. Across all cancer types, its incidence now ranks second worldwide, surpassed only by lung cancer. Early diagnosis and timely intervention are essential for improving patient prognosis and reducing breast cancer mortality [2]. Among current diagnostic modalities, ultrasound imaging is widely used in practice due to its real-time acquisition, non-invasiveness, lack of radiation exposure, and low cost [3]. Compared with imaging methods such as mammography that involve ionizing radiation, ultrasound examination has higher repeatability and is more acceptable to patients. It is particularly suitable for the screening of dense breast tissue. Moreover, ultrasound equipment is portable and can be operated bedside, which has unique advantages in primary medical care and areas with limited resources. With the development of portable ultrasound and artificial intelligence-assisted diagnostic technologies, the application value of ultrasound in the early screening of breast cancer has further increased. In particular, multimodal ultrasound imaging, including B-mode ultrasound, color Doppler flow imaging (CDFI) and ultrasound elastography (UE), provides complementary information on lesion morphology, vascularity, and tissue stiffness [4,5], as shown in Figure 1. Despite these advantages, effectively integrating heterogeneous information across modalities remains a challenging task. The differences in imaging mechanisms from different modalities often lead to inconsistent feature distributions [6], while existing methods tend to rely on simple weighted summation or concatenation to achieve cross-modal feature fusion [7]. Furthermore, medical imaging data is often affected by noise and incomplete modal acquisition, which further increases the difficulty [8]. These limitations highlight the necessity of designing a multimodal learning framework to fully utilize the complementary information among various modalities and adapt to imaging data with significant sample differences.

With the continuous development of deep learning, breast ultrasound diagnosis methods have attracted wide attention [9]. However, these existing methods mostly rely on a single strategy for missing modality handling, which is difficult to adapt to various missing scenarios with limited robustness [10]. To improve the accuracy of diagnosis, multi-modal medical image analysis has shown progress [11,12,13]. For instance, Wang et al. [14] proposed FESCA, which realizes effective fusion of pathological images and genomic data through feature enhancement and semantic alignment strategies. Misra et al. [15] constructed a weighted multi-modal U-Net and achieved synergistic optimization in the joint segmentation and classification of BUS and elastography ultrasound. Yan et al. [16] proposed TDFNet, which introduces contrastive clustering loss and invertible neural networks, showing promising performance in dynamic feature fusion and missing modality recovery. Although the existing methods have made significant progress, there are still obvious shortcomings. Most existing methods adopted a separate architecture with single-modal feature extraction followed by late fusion, where information interaction between modalities only occurs at the decision level, making it difficult to achieve mutual guidance and complementarity in the early stage of feature learning [17]. Meanwhile, existing methods lack targeted enhancement of key diagnostic regions in ultrasound images, such as lesion edges, blood-rich areas, and abnormal stiff areas, leading to insufficient distinguishability and stability of the model in feature representation [18]. In the field of multimodal breast ultrasound analysis, some current studies employ the Visual Transformer (ViT) as the backbone network for feature extraction and fusion [19,20,21]. However, ViT suffers from a large number of parameters and high inference latency, making it challenging to deploy in resource-constrained environments [22].

To address the above limitations, we proposed an efficient Cross-Modal Interaction and Dynamic Fusion Network (CIDFNet). The model utilizes an InceptionV3 backbone, which is lighter than ViT, as the shared backbone to extract basic multi-modal features, and then enhances key single-modal regions via the multi-scale feature enhancement module. The cross-attention interaction module is then designed to achieve early and deep information interaction among modalities. Finally, the dynamic fusion module and invertible neural network collaboratively complete uncertainty-weighted fusion and missing modality recovery. These modules work together to strike a balance between model accuracy and computational cost. Extensive experiments have presented that the proposed method obtains a reasonable balance between performance and computational cost, while exhibiting only limited robustness under simulated challenging conditions. The main contributions of this paper are summarized as follows:

1. We proposed CIDFNet, a unified framework that integrates multi-scale feature enhancement and cross-modal interaction for multimodal breast ultrasound diagnosis. The proposed model obtains a competitive performance while substantially reducing computational cost, reflecting a trade-off between accuracy and efficiency.
2. We introduced a cross-attention interaction module that combines a trusted dynamic fusion module and an invertible neural network for feature-adaptive fusion and reconstruction, enabling cross-modal integration.
3. Extensive experiments suggest that CIDFNet shows a trade-off between performance and computational cost, though its robustness is limited under simulated varying Gaussian noise conditions.

## 2. Related Work

In recent years, multimodal breast ultrasound diagnosis based on deep learning has attracted widespread attention due to its ability to effectively integrate complementary information from different imaging modalities [23]. This section systematically reviews the existing research from three aspects: multimodal fusion strategies, cross-modal interaction mechanisms, and network design with reduced computational cost. Firstly, it reviews the technological evolution of decision-level, feature-level, and adaptive fusion paradigms. Secondly, it analyzes the application status of the attention mechanism in cross-modal feature interaction [24]. Finally, it summarizes computationally efficient model architectures suitable for medical imaging scenarios [25]. Through this review, it can be observed that when adapting to the joint diagnosis of BUS, CDFI, and UE modalities, the existing methods adopt a paradigm of extracting independently and then fusing. As a result, the cross-modal complementary information fails to interact fully. Additionally, the imaging characteristics of the three modalities are significantly different, which further increases the difficulty of feature alignment and fusion, and the overall model’s computational efficiency requires further improvement.

### 2.1. Multi-Modal Learning

In the field of medical image analysis, deep learning has made significant progress and demonstrated superior performance in various tasks, including nodule recognition [26], tumor segmentation [27], lesion grading [28], and mammography image classification [29]. However, most existing studies are based on single-modal data, which makes it difficult to describe the multi-dimensional features of complex lesions, thus limiting further improvement in analysis performance. In contrast, multi-modal imaging provides complementary information from different imaging mechanisms, which has advantages in improving lesion representation. Therefore, intelligent cancer analysis methods based on multi-modal data have attracted increasing attention due to their significant potential in assisting decision-making [30,31,32]. Building upon the advantages of multi-modal imaging, a critical challenge lies in how to effectively integrate heterogeneous information from different modalities. To this end, multi-modal fusion strategies have evolved into three representative technical paradigms.

The first type is decision-level fusion [33], which independently infers for each modality and combines outputs through statistical rules or voting mechanisms. Although this method is easy to implement, it cannot establish deep cross-modal feature correlations, resulting in limited fusion capabilities. The second paradigm is feature-level fusion [6], where multi-modal features are connected or weighted in the middle layers of the network. Compared to decision-level fusion, it provides stronger representation capabilities. However, it often struggles to handle modal heterogeneity, resulting in inconsistent features, redundancy, and potential conflicts. The third paradigm is adaptive fusion [16], which dynamically learns modality-specific weights and relationships between modalities to achieve more flexible and robust feature integration. By adjusting the contribution of each modality according to different lesion characteristics and imaging patterns, this method has become the main strategy for multimodal breast ultrasound diagnosis, and has shown good adaptability under various image conditions. For example, Huang et al. [34] proposed the AW3M framework, which integrates three-modal features through a self-weighting strategy. Fang et al. [35] introduced Swin Transformer into multi-modal ultrasound diagnosis, using shifted window attention to capture cross-modal interactions. Mo et al. [36] developed HoVer-Trans, which combines anatomical prior knowledge for diagnosis without ROI annotation. TDFNet [16] further proposed a dynamic feature fusion module, in which modal weights are adjusted based on uncertainty estimated via a Dirichlet distribution to improve fusion robustness. Despite these advances, most existing methods still follow the paradigm of independent feature extraction and late-stage fusion. Therefore, cross-modal interactions are often delayed, and supplementary information in the early stages is not fully utilized. This limitation will weaken the model’s ability to handle image noise and differences in lesion size. It is prone to exhibiting fluctuating classification results on test samples with significant sample variations.

### 2.2. Cross-Modal Interaction and Fusion

Although the aforementioned multi-modal fusion strategies have shown promising performances, a fundamental challenge remains in effectively modeling cross-modal interactions. In particular, the discriminability of single-modal representations largely determines the upper bound of fusion performance. Therefore, the attention mechanism is an important approach for enhancing significant features and facilitating cross-modal information interaction.

In the area of single-modal feature enhancement, Hu et al. [37] introduced SENet, which adopts a multi-crop fusion strategy and recalibrates the channel-level features through compression and excitation mechanisms. However, this work used ResNet as the backbone and only focused on single-modal optimization, without involving cross-modal interaction. Roy et al. [38] further proposed the scSE module, which incorporates concurrent spatial and channel recalibration into the segmentation network via a block fusion strategy. However, despite employing SD-Net as the backbone, this approach lacks a dedicated mechanism to handle missing modalities. Cai et al. [39] developed CGDMNet, which utilizes the transformer fusion strategy and achieves cross-modal interaction in the Intermediate stage, using MAL as the backbone network. Liu et al. [40] proposed LHNet, which employs information fusion and multi-scale sliding window attention to balance long-range dependencies and computational efficiency. However, this method relies on a ViT backbone and performs fusion at the Late stage. All these methods did not consider the scenario of missing modalities, and the ViT-like architecture has high computational overhead.

To address the issue of cross-modal interaction, several studies [24,41,42] have explored fusion methods based on the attention mechanism. Ghantasala et al. [24] proposed HXM-Net, which adopts a Transformer fusion strategy and uses ViT as the backbone to complete cross-modal interaction at the Intermediate stage. It integrates multimodal breast ultrasound images, utilizes transfer learning to enhance the generalization ability across datasets, and combines interpretable artificial intelligence to improve the interpretability of clinical decisions. Dong et al. [41] developed DCFAN, which adopts a channel fusion strategy and uses CNNs as the backbone to complete cross-modal interaction at the Intermediate stage, integrating spatial features of B-mode ultrasound and shear wave elastography hardness features. Xu et al. [42] introduced MSFT-Net, which adopts SCAM fusion strategy and uses TimeSformer as the backbone to achieve feature aggregation at the Intermediate stage, improving the fusion efficiency by selectively retaining meaningful query-key interactions. Mondol et al. [43] designed KGCML network, which adopts MM-CAF fusion strategy, using ResNet50 as the backbone to achieve Hybrid dual-stage fusion, combining H-scan, Nakagami parameter images, and synthetic breast ultrasound images. Qian et al. [44] developed BMU-Net, which adopts Transformer fusion strategy and uses ResNet18 as the backbone to achieve Hybrid fusion as well, using Random Masking Training to handle the problem of incomplete modalities. Yan et al. [16] proposed TDF-Net, which adopts TDFM dynamic weighted fusion strategy, using ResNet50 as the backbone to complete fusion at the Intermediate stage, and for the first time introduced an invertible neural network (INN) to achieve feature-level reconstruction of missing modalities. When directly transferred to the BUS-CDFI-UE trimodal task and constraining the model parameter scale, these methods generally suffer from insufficient adaptability and poor computational efficiency.

Table 1 summarizes the differences between existing methods and the proposed CIDFNet from five aspects: fusion strategy, interaction timing, backbone network, computational cost, and missing modality handling. The early methods SENet and scSE only focus on single-modal optimization without cross-modal interaction. CGDMNet and LHNet adopt intermediate and late fusion, respectively, but their interaction stages are too late to establish effective feature correlations in shallow networks. HXM-Net, DCFAN, and MSFT-Net achieve cross-modal interaction at the intermediate level and outperform single-modal approaches. However, HXM-Net and LHNet employ ViT-based backbones, resulting in higher computational costs. KGCML adopts a hybrid fusion strategy that combines early and intermediate fusion, which enhances interaction capability but does not handle missing modalities. BMU-Net uses random masking training to handle missing modalities, but it does not perform feature-level reconstruction. TDF-Net is the first to introduce invertible neural networks for missing modality reconstruction. However, its backbone is ResNet50 with FLOPs as high as 364.882 G, leading to high deployment costs. In contrast, CIDFNet adopts a hybrid fusion strategy, achieving early-intermediate cross-stage feature interaction. It adopts InceptionV3 as the backbone and also employs invertible neural networks to restore missing modalities. With only 49.51 M parameters and 79.789 G FLOPs, CIDFNet strikes a trade-off among AUC, accuracy, and computational cost.

### 2.3. Lightweight Network

Optimizing the overall performance of multimodal breast ultrasound models requires not only cross-modal interaction but also computational efficiency. Consequently, various lightweight network architectures have been developed. The MobileNet series [45,46] reduces computational cost via depthwise separable convolutions while maintaining feature extraction capability. Xie et al. [47] proposed a multi-scale feature fusion shuffle network, utilizing dilated convolution to capture diverse features, with its core components built upon ShuffleNetV2 units. Choi et al. [48] enhanced the U-Net encoder with an Inception architecture to address feature extraction deficiencies in breast ultrasound segmentation.

For breast ultrasound applications, several task-specific lightweight designs have also been explored. Chen et al. proposed GDUNet [49], which adapts to limited computational resources by simplifying network structures and freezing shallow layers. Wu et al. introduced UltraLight VM-UNet [50], which balances diagnostic accuracy and efficiency through parallel lightweight branches. HAAU-Net [27] combined multi-scale adaptive attention with spatial-channel attention to enable efficient, real-time segmentation with low computational cost, which can be deployed on standard clinical equipment. These studies provide valuable insights for lightweight backbone design in medical imaging. However, most existing methods are mainly designed for bimodal inputs, making them difficult to directly adapt to the joint feature modeling across BUS, CDFI, and UE modalities. Moreover, the general lightweight backbone lacks optimization for the low contrast and multiple lesion features of breast ultrasound images, often resulting in insufficient feature extraction and compromised multi-modal fusion.

## 3. Materials and Methods

This section systematically introduces the dataset composition, experimental setup, evaluation metrics, and network architecture of CIDFNet.

### 3.1. Datasets

The multimodal breast ultrasound dataset used in this study was collected from the Second Affiliated Hospital of Harbin Medical University. It was originally established by Yan et al. [16] and has been widely applied to breast cancer diagnosis research. The dataset contains 1532 breast ultrasound images from 248 patients, covering three modalities: BUS, CDFI, and UE. The age of the patients ranges from 14 to 85 years, with an average age of 46 years. Among them, 145 cases are benign, and 103 are malignant, with all diagnoses confirmed by pathological examination. Fibroadenoma constitutes the majority of benign lesions, whereas invasive ductal carcinoma is the most prevalent malignant type. All images were acquired by experienced radiologists using high-end ultrasound devices such as Philips and Siemens. In this work, the dataset is randomly divided at the patient level into training, validation, and test sets with a ratio of 60%, 20%, and 20%, respectively, to avoid data leakage. All images are resized to a unified resolution of 224×224 pixels.

### 3.2. Implementation Details

All experiments were implemented using the PyTorch 2.4.1 framework on a single NVIDIA GeForce RTX 4090 GPU (NVIDIA Corporation, Santa Clara, CA, USA). The backbone is initialized with InceptionV3 pre-trained on the ImageNet dataset. To facilitate convergence and adapt to the characteristics of breast ultrasound images, the parameters of the first eight layers are frozen during training. The pre-trained weights are loaded to further stabilize training. All hyperparameters are tuned and determined based on this fixed validation set. The model is trained for 150 epochs with a batch size of 4, an initial learning rate of $2\times 10^{-5}$, and a weight decay coefficient of $1\times 10^{-5}$. The Adam optimizer is employed, and the learning rate is kept constant throughout training to ensure stable optimization. For reproducibility, the random seed is fixed at 42.

To mitigate overfitting, several data augmentation strategies are applied during training, including random horizontal and vertical flips, random brightness and contrast perturbations, and random Gaussian blur. During validation and testing, only resizing and center cropping are performed to ensure consistent evaluation. In addition, all modality images are standardized to have zero mean and unit variance, reducing distribution discrepancies across modalities. During the testing phase, inference is performed with a batch size of 1, and a uniform decision threshold of 0.5 is applied. All ablation studies, comparative experiments, and noise perturbations utilize the fixed test set and are evaluated by loading the best model weights saved during the training process of each model. The entire experimental configuration remains consistent to ensure fair and comparable results.

### 3.3. Evaluation Metrics

To comprehensively evaluate the proposed method, five widely used metrics are adopted, including the Area Under Curve (AUC), accuracy, recall, precision, and F1-score. Their definitions are given as follows:

(1) $$\begin{array}{cc} \mathrm{accuracy} & =\frac{\mathrm{TP}+\mathrm{TN}}{\mathrm{TP}+\mathrm{TN}+\mathrm{FP}+\mathrm{FN}}, \end{array}$$

(2) $$\begin{array}{cc} \mathrm{recall} & =\frac{\mathrm{TP}}{\mathrm{TP}+\mathrm{FN}}, \end{array}$$

(3) $$\begin{array}{cc} \mathrm{precision} & =\frac{\mathrm{TP}}{\mathrm{TP}+\mathrm{FP}}, \end{array}$$

(4) $$\begin{array}{cc} F1-\mathrm{score} & =\frac{2\times \mathrm{precision}\times \mathrm{recall}}{\mathrm{precision}+\mathrm{recall}}, \end{array}$$

where TP, FP, FN, and TN denote the numbers of true positives, false positives, false negatives, and true negatives, respectively. The AUC is computed based on the receiver operating characteristic (ROC) curve, which reflects the trade-off between true positive rate and false positive rate across different decision thresholds.

### 3.4. Overall Framework

**Figure 2:**
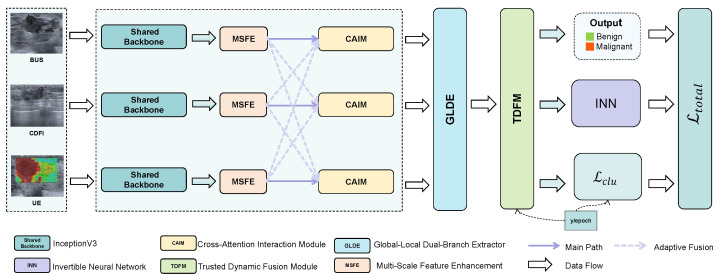
Overall framework of the proposed CIDFNet.

To utilize the complementary information across modalities, we proposed CIDFNet, an efficient multimodal learning framework for breast ultrasound diagnosis. As shown in Figure 2, CIDFNet adopts a progressive pipeline that integrates feature enhancement, cross-modal interaction, and adaptive fusion. It consists of six modules: a shared backbone network, a multi-scale feature enhancement module (MSFE), a cross-attention interaction module (CAIM), a global-local dual-branch feature extractor (GLDE), a trusted dynamic fusion module (TDFM), and an invertible neural network (INN) for missing modality restoration. Specifically, the GLDE, TDFM, and INN adopt the established architecture from TDFNet [16], which have proven effective in dynamic feature fusion and missing modality recovery. Building upon this, the main innovations of this proposed model are threefold: Firstly, the proposed MSFE and CAIM enable fine-grained single-modal feature enhancement and early cross-modal interaction, respectively. Secondly, the backbone network is optimized by replacing the original ResNet50 with InceptionV3 to reduce computational overhead. Thirdly, taking into account the specific characteristics of multimodal breast ultrasound, these modules have been jointly optimized to maintain the original advantages while achieving a good balance between performance and computational cost.

Given multimodal ultrasound inputs, a shared backbone is first employed to extract modality-specific feature representations with consistent dimensions. These features are then enhanced by the MSFE module, which is designed to improve discriminative regions. The enhanced features are subsequently fed into the CAIM module, where early-stage cross-modal interaction is performed to capture complementary information across modalities. To further improve representation capability, the GLDE module jointly models global structural patterns and local fine-grained details. Finally, the TDFM module adaptively integrates modality-specific features by estimating their relative reliability, producing a robust fused representation for classification. During training, the INN-based reconstruction mechanism proposed in TDFNet [16] is introduced. Random modality masking is applied as a regularization strategy, forcing the INN to learn accurate cross-modal mappings and enhancing feature alignment between modalities.

### 3.5. Shared Backbone

To balance feature representation capability and computational efficiency, we adopted InceptionV3 pre-trained on ImageNet as the shared backbone. Benefiting from its multi-branch design and factorized convolutions, InceptionV3 can capture multi-scale features while maintaining a relatively low computational cost.

To adapt the backbone for medical imaging tasks, its auxiliary classifier branch is removed, and the parameters of the first eight layers are frozen to preserve general low-level features and stabilize training. All modalities share the same backbone weights to ensure consistent feature extraction. The backbone outputs feature maps with 768 channels, which are then reduced to 512 channels via a 1×1 convolution. This operation not only reduces computational overhead but also enables cross-channel feature interaction. Subsequently, an adaptive average pooling layer is applied to yield feature maps of 28×28, ensuring consistent spatial resolution across inputs. The resulting features are denoted as $f_{m}^{\left(0\right)}\in R^{B\times 512\times 28\times 28}$.

### 3.6. Multi-Scale Feature Enhancement Module

The quality of single-modality features often affects subsequent fusion. However, breast ultrasound images have significant heterogeneity and multi-scale lesion features. To address this, we proposed the MSFE module to enhance feature representation.

**Figure 3:**
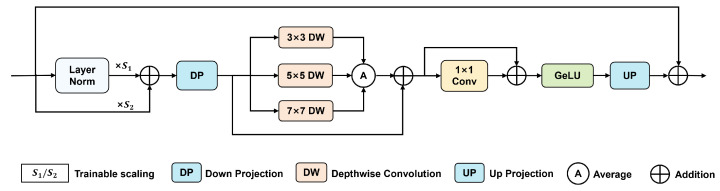
Architecture of the Multi-Scale Feature Enhancement (MSFE) module.

As shown in Figure 3, given the input features $X\in R^{B\times N\times C}$, where *B* is the batch size, *N* is the feature sequence length, and *C* is the number of channels, we first performed adaptive feature modulation:

(5) $$X_{\mathrm{mod}}=\mathrm{LN}\left(X\right)\cdot S_{1}+X\cdot S_{2},$$

where LN denotes layer normalization, which is applied to the feature sequence dimension to adjust the feature distribution of each channel to a standard distribution, alleviating feature distribution shift. $S_{1}$ and $S_{2}$ are trainable scaling parameters, which perform channel-wise modulation on normalized features and original features, respectively. This design balances normalized and original features, improving feature stability and cross-modal distribution alignment.

The modulated features are then linearly projected and reshaped into spatial feature maps, and this process can be formulated as follows:

(6) $$X_{\mathrm{sp}}=\mathrm{Reshape}\left(\mathrm{Linear}\left(X_{\mathrm{mod}}\right),\;\left(B,C^{\prime },H,W\right)\right),$$

where $C^{\prime }$ is the number of output channels of the linear layer and $H=W=\sqrt{N}$, requiring the original feature sequence length *N* to be a perfect square, which can be satisfied by adaptive pooling after the backbone output.

To capture multi-scale spatial features, three parallel depthwise convolution branches with kernel sizes 3×3, 5×5, and 7×7 are employed to model fine-grained textures, structural patterns, and global morphology, respectively. Their outputs are averaged and fused, combined with residual connections to reduce information loss during multi-scale fusion. By assigning identical weights to features across all scales, the average fusion strategy mitigates the risk of overfitting caused by the introduction of additional learnable parameters. The residual connection preserves original spatial details and alleviates information loss during multi-scale feature aggregation. The process is summarized as follows:

(7) $$F_{\mathrm{agg}}=\frac{{\mathrm{DWConv}}_{3\times 3}\left(X_{\mathrm{sp}}\right)+{\mathrm{DWConv}}_{5\times 5}\left(X_{\mathrm{sp}}\right)+{\mathrm{DWConv}}_{7\times 7}\left(X_{\mathrm{sp}}\right)}{3}+X_{\mathrm{sp}}.$$

Finally, the enhanced features are projected back using a linear transformation and residual projection:

(8) $$X_{\mathrm{out}}=X+\mathrm{Linear}\left(F_{\mathrm{agg}}\right).$$

This design enhances discriminative features while preserving original semantic information, providing inputs for subsequent cross-modal interaction.

### 3.7. Cross-Attention Interaction Module

In multimodal breast ultrasound diagnosis, single-modal features have limited information coverage and can hardly describe the multidimensional characteristics of lesions comprehensively. To enable information interaction across modalities, we introduced a cross-attention interaction module (CAIM). Unlike late fusion, this module performs early-stage interaction through cross-attention, allowing each modality to incorporate complementary information from others, achieving adaptive information interaction and feature enhancement across modalities through the multi-head attention mechanism.

**Figure 4:**
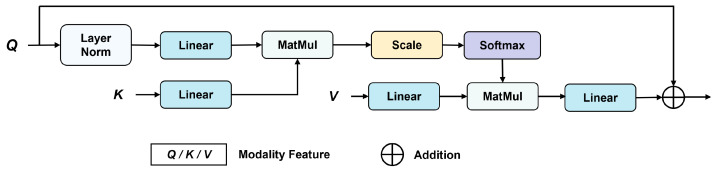
Architecture of the Cross-Attention Interaction Module (CAIM).

As illustrated in Figure 4, for a given modality, its feature is treated as the query *Q*, while the features from other modalities are concatenated to form the key *K* and value *V*. The module first performs layer normalization on the query features *Q* to stabilize the training process. Subsequently, the features *Q*, *K*, and *V* are linearly projected into the attention calculation space and input into the multi-head attention mechanism, enabling the model to learn cross-modal correlation and diversity from different modality features, further enhancing the learning ability and expressiveness of the model. The correlation between *Q* and *K* is calculated via scaled dot-product, and a scaling factor is introduced to mitigate numerical instability caused by high-dimensional feature representations, ensuring stable attention estimation. Then, the correlation matrix is normalized via softmax to produce attention weights, which are used to aggregate the value features *V*, allowing the model to selectively incorporate informative cues from other modalities while suppressing noise, and the core formula is shown as follows:

(9) $$\mathrm{Attn}\left(Q,K,V\right)=\mathrm{softmax}\left(\frac{QK^{⊤}}{\sqrt{d_{k}}}\right)V,$$

where $d_{k}$ refers to the feature dimension of a single attention head.

The aggregated features are first projected back to the original feature dimension via a linear transformation. A residual connection is then introduced by adding the projected features to the query feature *Q*, yielding the final enhanced representation. The core formulation is given as follows:

(10) $$F_{\mathrm{out}}=Q+\mathrm{Linear}\left(\mathrm{Attn}(Q,K,V)\right).$$

Through this design, each modality retains its intrinsic characteristics while integrating complementary features from other modalities, resulting in more informative and feature representations.

### 3.8. Multi-Task Joint Loss

To collaboratively optimize classification performance, cross-modal consistency, and feature alignment, we introduced a multi-task joint loss function consisting of contrastive clustering loss, modality transformation loss, and uncertainty-aware fusion loss, and the total loss is defined as follows:

(11) $$L_{\mathrm{total}}=\omega_{\mathrm{clu}}L_{\mathrm{clu}}+\omega_{\mathrm{mod}}L_{\mathrm{mod}}+\omega_{\mathrm{uaf}}L_{\mathrm{uaf}},$$

where $\omega_{\mathrm{clu}}$, $\omega_{\mathrm{mod}}$, and $\omega_{\mathrm{uaf}}$ are weight coefficients to balance the impact of different task objectives on model training. These coefficients are empirically determined via grid search on the validation set from the fixed data split, and are set to 0.5, 2.5, and 1.5, respectively.

The contrastive clustering loss $L_{\mathrm{clu}}$ is designed to enforce the semantic consistency across modalities while preserving class-level discriminability. Given that different modalities of the same patient describe a shared pathological entity, their representations are expected to be aligned in the latent space despite appearance heterogeneity. This loss consists of a supervised clustering term and an unsupervised alignment term:

(12) $$L_{\mathrm{clu}}=\frac{\alpha }{2}L_{s}+\alpha L_{u},$$

where α is a balancing coefficient set to 0.5. The supervised clustering loss $L_{s}$ uses learnable cluster centers *C* to pull the features of each modality closer to the cluster centers of their corresponding categories, which can be formulated as:

(13) $$L_{s}=-\frac{1}{MN}\sum_{i=1}^{M}\sum_{j=1}^{N}\left[y_{j}\log\left(p_{ij}\right)+\left(1-y_{j}\right)\log\left(1-p_{ij}\right)\right],$$

where *M* and *N* denote the number of modalities and samples, respectively. $y_{j}$ is the true label of the *j*-th sample, and $p_{ij}=\mathrm{Softmax}\left(Cf_{i,j}\right)$ represents the predicted probability of the *j*-th sample in the *i*-th modality. To further enforce cross-modal consistency, an unsupervised clustering loss based on Sinkhorn–Knopp normalization [51] was introduced to exchange clustering prediction logic between different modalities, and the formula is defined as follows:

(14) $$L_{u}=-\frac{1}{MN}\sum_{j=1}^{N}\left[q_{1j}\log\left(p_{2j}\right)+q_{2j}\log\left(p_{3j}\right)+q_{3j}\log\left(p_{1j}\right)\right],$$

where $q_{i,j}$ denotes the soft cluster assignment generated by Sinkhorn normalization.

To facilitate cross-modal feature alignment, we introduced a modality transformation loss $L_{\mathrm{mod}}$, which employs the Kullback–Leibler (KL) divergence to measure the difference between the feature distribution generated by the INN and the ground-truth feature distribution. During training, random modality masking is applied as a regularization strategy, forcing the INN to learn robust cross-modal mappings and enhancing feature alignment between modalities. The loss is defined as follows:

(15) $$L_{\mathrm{mod}}=\sum_{m=1}^{M}\sum_{A\subset M\setminus \{m\}}\mathrm{KL}\left(f_{m}^{\mathrm{gt}}\|{\hat{f}}_{m}^{A}\right),$$

where M={BUS,CDFI,UE} denotes the set of modalities, *A* is the subset of available modalities, $f_{m}^{\mathrm{gt}}$ represents the ground-truth feature of the missing modality *m*, ${\hat{f}}_{m}^{A}$ is the feature recovered by the INN from the available modalities, and the calculation form of the KL divergence is defined as follows:

(16) $$\mathrm{KL}\left(P\|Q\right)=\sum_{k=1}^{K}P\left(k\right)\log\frac{P(k)}{Q(k)}.$$

The uncertainty-aware fusion loss $L_{\mathrm{uaf}}$ is designed to jointly optimize classification performance and predictive reliability. Specifically, this loss integrates the classification cross-entropy and the evidence regularization term, with its formula as follows:

(17) $$L_{\mathrm{uaf}}=-\frac{1}{N}\sum_{i=1}^{N}\sum_{k=1}^{K}y_{ik}\log p_{ik}+\lambda \sum_{i=1}^{N}\frac{K}{S_{i}},$$

where K=2 denotes the number of classes, $y_{ik}$ is the one-hot label of the *i*-th sample, and $p_{ik}$ is the predicted class probability output by the TDFM. In the evidence regularization term, $S_{i}=\sum_{k=1}^{K}\alpha_{ik}$ represents the Dirichlet strength, and $\alpha_{ik}$ is the evidence value of the *i*-th sample for the *k*-th class. Additionally, $\frac{K}{S_{i}}$ reflects the uncertainty of the prediction. The regularization term penalizes low-evidence predictions, thereby discouraging overconfident outputs and improving uncertainty calibration. The regularization coefficient λ is experimentally determined to be set to 0.1, which is used to balance the weight between classification accuracy and uncertainty estimation.

## 4. Results

This section reports the experimental results of CIDFNet, including classification performance, computational efficiency, and behavior under Gaussian noise perturbations. The evaluation is conducted through ablation studies, comparative experiments, and robustness analysis.

### 4.1. Ablation Study

To investigate the contribution of each core component to both performance and computational efficiency, we conducted a comprehensive ablation study based on the TDFNet baseline. The evaluation was performed from three aspects, including performance, parameter count, and computational complexity, and the results are summarized in Table 2.

As shown in the first row, the baseline model reported an AUC of 86.72% and a precision of 80.00%, but it required 55.70 M parameters and 364.882 G FLOPs, which implies its relatively high computational overhead may restrict deployment on low-power hardware. Introducing the MSFE module independently, as shown in the second row, accuracy improved from 77.55% to 83.67%, and both recall and F1-score increased to 70.00% and 77.78%, respectively, while the AUC slightly decreased to 80.00%. This result indicated that MSFE can capture discriminative lesion features at the early stage. To reduce the computational complexity of the model, we replaced the original backbone with InceptionV3 while keeping other components unchanged. Although most classification metrics decreased compared with the baseline, the model still obtained a competitive AUC of 81.20%. More importantly, the number of parameters reduced from 55.70 M to 48.23 M, and FLOPs decreased dramatically from 364.882 G to 75.904 G, demonstrating a substantial improvement in computational efficiency with an acceptable performance trade-off. When the CAIM module was introduced independently, the recall improved to 70.00%, highlighting the benefit of cross-modal interaction in capturing complementary information. However, this improvement is accompanied by increased parameter count and computational cost. When combining the backbone InceptionV3 with either MSFE or CAIM, both parameters and computational cost were substantially reduced. However, the decline in classification performance indicates that the reduced capacity of the lightweight backbone is insufficient to sustain the feature enhancement and interaction modules. Similarly, integrating MSFE and CAIM without the lightweight backbone fails to yield improvements and incurs additional computational overhead.

In contrast, the complete integration of MSFE, InceptionV3, and CAIM reported an AUC of 85.69% with parameters of 49.51 M and FLOPs of 79.789 G. Compared to the baseline, CIDFNet reduces computational cost by 78.13% while improving precision by 3.33%. Although the AUC is marginally lower than the baseline, the overall results suggest that CIDFNet obtains a trade-off. These findings suggest the potential usefulness of the proposed components and the necessity of their collaborative design.

Furthermore, to evaluate the trade-off reported by the proposed InceptionV3-based framework, we compared it with five mainstream lightweight alternatives: Mobilenetv3-small [52], EfficientNet-B0 [53], ShuffleNetV2 [54], GhostNet [55], and ConvNeXt-Tiny [56].

As shown in Table 3, the proposed method reports a higher AUC compared with the evaluated lightweight backbones. Specifically, while ConvNeXt-Tiny exhibits competitive performance, its parameter quantity and computational cost are higher than those of InceptionV3. Conversely, although Mobilenetv3-small, EfficientNet-B0, ShuffleNetV2, and GhostNet reduce parameters, they suffer from degradation in both AUC and precision. These comparisons suggest that InceptionV3 provides a trade-off between accuracy and computational efficiency for the multimodal breast ultrasound task.

### 4.2. Comparison Results

To evaluate the performance of CIDFNet, we compared it with four representative multimodal learning methods, including TDFNet [16], BINDS [57], FusionM4Net [58], and MSMFN [59]. To ensure fairness, all methods strictly followed the network architectures reported in their original papers.

As shown in Table 4, CIDFNet shows competitive performance on the multimodal breast ultrasound dataset, with an AUC of 85.69%, an accuracy of 75.51%, a recall rate of 50.00%, an F1 score of 62.50%, and a precision rate of 83.33%. Compared with the baseline TDFNet, CIDFNet improves precision by 3.33%, while reducing parameters by 11.11% and FLOPs by 78.13%. BINDS achieves a comparable AUC of 85.00%, but its lower precision and larger model size may affect its suitability for resource-constrained settings. In contrast, while FusionM4Net and MSMFN reduce computational overhead, they compromise performance. Notably, FusionM4Net yields an AUC of merely 48.79% and a precision of 50%, and MSMFN achieves an AUC of 76.55% with the same precision. This suggests that aggressive model simplification may lead to performance degradation in multimodal breast ultrasound tasks.

Overall, the comparison experiments indicate that CIDFNet balances performance between precision and computational efficiency in multimodal breast ultrasound. It yields relatively competitive AUC and precision compared to representative multimodal methods, while requiring fewer parameters and lower computational overhead than TDFNet and BINDS. Although FusionM4Net and MSMFN are lighter, their accuracy degradation offsets their computational advantages.

### 4.3. Robustness Analysis

Multimodal breast ultrasound images are inevitably affected by random noise due to device conditions, operator variability, and tissue scattering. Such noise obscures discriminative features of lesion regions, posing a challenge to analysis stability. To evaluate the robustness of the proposed CIDFNet, we injected Gaussian noise into the raw images under two settings: single-modality perturbation and full-modality perturbation. It should be noted that the Gaussian noise serves only as a simplified perturbation to observe the model’s performance variations, and it cannot substitute for the authentic noise and complex artifacts inherent in ultrasound imaging.

As summarized in Table 5, in the single-modality perturbation setting corresponding to the first three rows, applying 10% Gaussian noise independently to a single modality (BUS, CDFI, or UE) yields minimal performance degradation. For instance, with the noise applied solely to the BUS modality, the model retains an AUC of 85.69% and an accuracy of 75.51%, which is comparable to the noise-free setting. When noise is only applied to the CDFI modality, the accuracy reaches 75.51%, indicating a degree of tolerance to noise perturbations. In the case of noise in the UE modality, the F1-score attains 62.50%. These results indicate that CIDFNet shows limited performance variation when a single modality is degraded. This behavior may be related to the dynamic fusion mechanism. By incorporating uncertainty modeling, the module adaptively down-weights the corrupted modality and emphasizes information from clean modalities, thereby helping to maintain overall performance.

In the full-modality perturbation setting corresponding to rows four through seven, Gaussian noise was simultaneously injected into all modalities at intensities ranging from 10% to 20%. Unsurprisingly, degrading all modalities simultaneously imposes a substantially greater challenge. Specifically, while performance exhibits moderate stability at 10% noise, increasing the intensity to 20% causes the accuracy to drop to 67.35% and AUC to 80.17%. Notably, recall exhibits a sharper decline from 50% to 30% than precision. This discrepancy can be attributed to the distinct characteristics of malignant lesions. Specifically, their inherently low-contrast and blurred boundaries become further obscured by background speckle under high-intensity noise, leading to increased missed detections. Conversely, the clearer morphological features of benign lesions are less affected, resulting in a smaller drop in precision.

**Figure 5:**
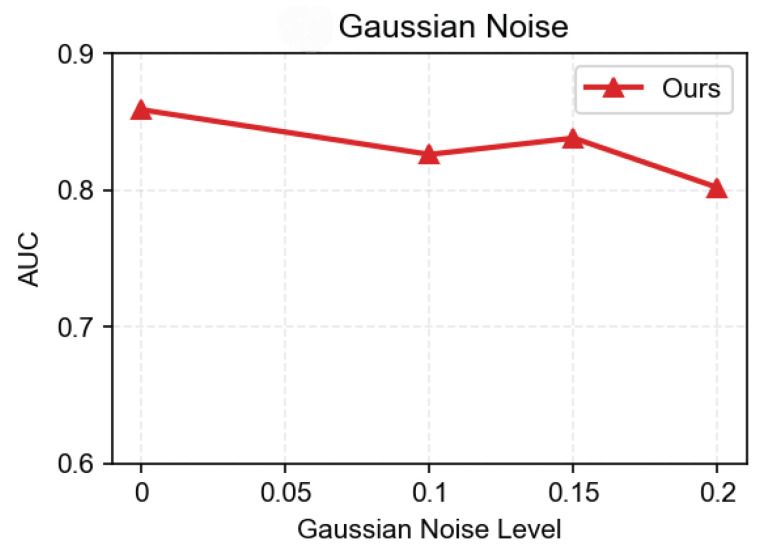
AUC variation curve of CIDFNet under gradually increased full-modal Gaussian noise.

Figure 5 further indicates the AUC decay curve of CIDFNet under full-modal Gaussian noise perturbation. The proposed CIDFNet exhibits a gradual degradation as noise increases from 0% to 20%, with the AUC declining from 85.69% to 80.17%. Although high-intensity noise leads to performance degradation, CIDFNet obtains an AUC above 80% even at 20% noise level, suggesting that the model maintains a certain level of discriminative performance under controlled noise conditions. These results suggest limited performance degradation under Gaussian noise, while performance varies across different noise levels.

## 5. Discussion

The proposed CIDFNet shows a trade-off between prediction performance and computational efficiency for multimodal breast ultrasound analysis. Compared with existing approaches, the model obtains a competitive classification performance while requiring fewer computational resources. This suggests that appropriately designed feature enhancement and cross-modal interaction mechanisms may support representation learning under constrained model capacity.

From the perspective of component analysis, the results of the ablation study indicate that each module contributes differently to the overall performance. The MSFE module enhances modality-specific feature representation, and its effect is mainly reflected in sensitivity-related metrics. The CAIM is associated with enhanced global feature alignment by enabling early-stage information exchange across modalities. In contrast, the use of an InceptionV3 backbone reduces computational cost, though it leads to some variation in performance compared with heavier architectures. When combined, these components exhibit complementary behavior, resulting in a trade-off between performance and efficiency.

In terms of comparison with other multimodal methods, CIDFNet presents competitive results in terms of AUC and precision while maintaining a relatively lower computational cost. However, it is also observed that methods with lower computational complexity may exhibit reduced classification performance, indicating that excessive simplification of model structure can affect feature representation capacity in multimodal settings.

The robustness analysis evaluates the model under synthetic Gaussian noise perturbations. Under single-modality perturbations, only minor performance variations are observed, suggesting that the dynamic fusion strategy may help reduce the impact of degraded inputs by leveraging complementary information from other modalities. However, when noise is applied to all modalities simultaneously, a more noticeable performance decline is observed, particularly in sensitivity-related metrics. It should be noted that the Gaussian noise used in this study represents a simplified simulation and does not fully reflect the complexity of real ultrasound imaging artifacts. Therefore, these results should be interpreted as an analysis of model behavior under controlled perturbations rather than as evidence of robustness in clinical or real-world scenarios.

In addition, the relatively moderate recall observed in the experiments reflects the influence of the current decision threshold and the inherent trade-off between sensitivity and precision under the adopted optimization setting. Since a fixed threshold is used for all evaluations without additional calibration, the model tends to produce more conservative outputs under the current threshold setting, which reduces false positives while also affecting the detection of some positive cases. This behavior should be interpreted in relation to the experimental configuration and task settings rather than as an isolated performance limitation.

Despite these findings, several limitations should be acknowledged. The study relies on a single-institution dataset without external validation, limiting generalizability. The Gaussian noise analysis serves only as a preliminary simulation and does not capture the full complexity of real-world ultrasound imaging. Additionally, the framework focuses primarily on the image data and does not incorporate clinical metadata. Future work will explore multi-center validation and integration of additional data sources to better assess real-world utility.

## 6. Conclusions

This study proposes CIDFNet, an efficient multimodal breast ultrasound diagnosis framework. It integrates an InceptionV3-based shared backbone, whose computational cost is lower than that of ResNet50, together with a multi-scale feature enhancement module and a cross-modal interaction module under a unified architecture. It is designed to address the challenges of heterogeneous information fusion, limited utilization of lesion features, and computational cost. Experimental results show that CIDFNet shows a trade-off between precision and computational cost in this setting. Future work will focus on integrating multi-center data and additional modalities to further promote the practical application.

## Figures and Tables

**Table 1:** Comparison of representative modal fusion methods.

Methods	Fusion Strategy	Interaction	Backbone	Parameters (M)	FLOPs (G)	Missing Modality Handling
SENet [37]	Multi-crop	None	ResNet	None	None	None
scSE [38]	scSE block	None	SD-Net	None	None	None
CGDMNet [39]	Transformer	Intermediate	MAL	25.06	6.37	None
LHNet [40]	Information	Late	ViT	2.95	33.15	None
HXM-Net [24]	Transformer	Intermediate	ViT	None	None	None
DCFAN [41]	Channel	Intermediate	CNNs	None	None	None
MSFT-Net [42]	SCAM	Intermediate	TimeSformer	None	None	None
KGCML [43]	MM-CAF	Hybrid	ResNet50	39.41	23.99	None
BMU-Net [44]	Transformer	Hybrid	ResNet18	None	None	Random Masking
TDF-Net [16]	TDFM	Intermediate	ResNet50	55.7	364.882	INN
Ours	CAIM+TDFM	Hybrid	InceptionV3	**49.51**	**79.789**	INN

**Table 2:** Ablation study of the main components of CIDFNet. Best results are in **bold**.

MSFE	InceptionV3	CAIM	AUC (%)	Accuracy (%)	Recall (%)	F1-Score (%)	Precision (%)	Parameters (M)	FLOPs (G)
×	×	×	**86.72**	77.55	60.00	68.57	80.00	55.70	364.882
✔	×	×	80.00	**83.67**	**70.00**	**77.78**	87.50	55.93	365.063
×	✔	×	81.20	71.43	55.00	61.11	68.75	**48.23**	**75.904**
×	×	✔	85.17	77.55	**70.00**	71.79	73.68	57.80	371.057
✔	✔	×	83.97	75.51	55.00	64.71	78.57	48.46	76.085
×	✔	✔	82.93	73.47	45.00	58.06	81.82	50.33	82.079
✔	×	✔	80.00	75.51	65.00	68.42	72.22	56.98	368.767
✔	✔	✔	85.69	75.51	50.00	62.50	**83.33**	49.51	79.789

In this table, ✔ indicates that the corresponding module is enabled, and × indicates that it is disabled.

**Table 3:** Comparative results of different lightweight backbones on the multimodal breast ultrasound dataset. Best results are in **bold**.

Methods	AUC (%)	Accuracy (%)	Recall (%)	F1-Score (%)	Precision (%)	Parameters (M)	FLOPs (G)
Mobilenetv3-small	72.59	71.43	60.00	63.16	66.67	**37.61**	42.459
EfficientNet-B0	71.38	69.39	**70.00**	65.12	60.87	44.14	48.865
ShuffleNetV2	67.24	65.31	60.00	58.54	57.14	38.50	44.128
GhostNet	65.52	55.10	15.00	21.43	37.50	38.06	**41.768**
ConvNeXt-Tiny	80.86	73.47	60.00	64.86	70.59	91.48	121.718
InceptionV3 (Ours)	**85.69**	**75.51**	50.00	**62.50**	**83.33**	49.51	79.789

**Table 4:** Comparative results of different methods on the multimodal breast ultrasound dataset. Best results are in **bold**.

Methods	AUC (%)	Accuracy (%)	Recall (%)	F1-Score (%)	Precision (%)	Parameters (M)	FLOPs (G)
TDFNet	**86.72**	**77.55**	**60.00**	**68.57**	80.00	55.70	364.882
BINDS	85.00	73.47	50.00	60.61	76.92	64.42	**27.451**
FusionM4Net	48.79	59.18	30.00	37.50	50.00	95.15	49.581
MSMFN	76.55	69.39	50.00	57.14	66.67	88.64	41.192
Ours	85.69	75.51	50.00	62.50	**83.33**	**49.51**	79.789

**Table 5:** Robustness experimental results of the proposed method on the multimodal breast ultrasound dataset with different noise levels.

Noise	BUS	CDFI	UE	AUC (%)	Accuracy (%)	Recall (%)	F1-Score (%)	Precision (%)
10%	✔	×	×	85.69	75.51	50.00	62.50	83.33
10%	×	✔	×	85.86	75.51	45.00	60.00	90.00
10%	×	×	✔	83.10	75.51	50.00	62.50	83.33
0%	✔	✔	✔	85.69	75.51	50.00	62.50	83.33
10%	✔	✔	✔	82.59	75.51	50.00	62.50	83.33
15%	✔	✔	✔	83.79	69.39	35.00	48.28	77.78
20%	✔	✔	✔	80.17	67.35	30.00	42.86	75.00

In this table, ✔ indicates that the corresponding modality is used, and × indicates that it is not used.

## Data Availability

A publicly available dataset was used in the article. https://www.kaggle.com/datasets/timesxy/multimodal-breast-ultrasound-dataset-us3m, accessed on 1 November 2025.
